# Association of Self-Reported Dietary and Drug Compliance with Optimal Metabolic Control in Patients with Type 2 Diabetes: Clinic-Based Single-Center Study in a Developing Country

**DOI:** 10.1155/2018/3421476

**Published:** 2018-07-24

**Authors:** Thilak Priyantha Weerarathna, Miyuru Kavinda Weerarathna, Vidarsha Senadheera, Herath Mudiyanselage Meththananda Herath, Gayani Liyanage

**Affiliations:** ^1^Faculty of Medicine, University of Ruhuna, Matara, Sri Lanka; ^2^Faculty of Medicine, University of Colombo, Colombo, Sri Lanka

## Abstract

**Introduction:**

Influence of dietary and drug compliance of patients with diabetes on attainment of optimal metabolic (glucose and lipid) control remains underexplored in developing countries.

**Materials and methods:**

Self-reported dietary and drug compliance score of 0–10, glycosylated hemoglobin (HbA1c), and low-density lipoprotein (LDL) levels were obtained from patients with diabetes. HbA1c <7% and LDL <100 mg/dL were used to define optimal glucose and lipid control, respectively. Proportions achieving each and both optimal parameters were estimated. Regression analysis was used to study the association of age, gender, age of onset and the duration of diabetes, self-reported dietary, and drug compliance scores with achievement of both parameters.

**Results:**

Mean (SD) age and duration of diabetes of 207 patients were 55 (10) and 10 (03) years. Optimal glucose and LDL control were achieved by 30% and 62%, and 23% had achieved both. Regression analysis revealed significant association of self-reported high dietary compliance with achievement of both targets.

**Conclusions:**

Findings highlight the suboptimal glucose and lipid control among patients with diabetes. Significant association of better dietary compliance with control of both parameters emphasizes the value of proper dietary adherence in achieving the optimal metabolic control among patients with diabetes.

## 1. Introduction

Type 2 diabetes (T2DM) is spreading as an epidemic in the developing countries including Sri Lanka, leading to substantial morbidity and mortality among the affected individuals [1]. Studies reveal that the incidence of most of the major complications of T2DM could be reduced or delayed by control of blood glucose, blood pressure, and adverse lipids [2, 3]. Findings of these studies emphasize that intensive control of not only blood glucose but also adverse lipids such as low-density lipoproteins (LDLs) and blood pressure is necessary to reduce the micro- and macrovascular complications of diabetes. Based on those studies, professional organizations have laid down the optimal levels of blood glucose, blood pressure, and adverse lipids that should be achieved in order to prevent and delay the onset of major complications of diabetes [4, 5].

However, audits and surveys conducted in different settings reveal that recommended blood glucose and lipid targets have not been achieved by a substantial proportion of patients with diabetes under treatment [6, 7]. Several factors such as gender, poor adherence to prescribed pharmacotherapy and diet, absence of symptoms or complications, and low socioeconomic class have been known to be associated with suboptimal control [8, 9].

Rising incidence of T2DM and associated cardiovascular disease (CVD) and other complications has been identified as emerging public health concerns in the developing countries. In order to control the dual epidemics of T2DM and CVD, healthcare authorities require information on the attainment of the recommended therapeutic goals of cardiometabolic risk factors and clinical and demographic associations of their suboptimal control. Such findings would enable the healthcare providers to identify and prioritize the risk factors which need more intensive control and also to recognize the vulnerable groups who need special attention. But, there is a paucity of research focused on this area in the developing countries including Sri Lanka. Only a single study reported on poor attainment of glycemic control measured by fasting blood glucose and it too failed to report on the clinical associations of suboptimal control [10]. There are no studies in the literature on attainment of recommended lipid goals among Sri Lankan patients with T2DM.

## 2. Objectives

We aimed to study the recommended therapeutic goal achievement with regard to two of the major cardiometabolic risk factors, namely, blood glucose and LDL cholesterol, in a sample of patients attending a diabetes outpatient clinic in southern Sri Lanka. We obtained information on laboratory reports done at the index visit. Control of glucose was assessed by HbA1c and lipids by LDL cholesterol assessment. We used the therapeutic goals recommended by the American Diabetes Association to categorize patients with optimal control of blood glucose (HbA1c <7%) and LDL cholesterol (LDL-C <100 mg/dL).

We also studied the association of factors such as gender, age at onset and the duration of diabetes, presence of established coronary artery disease, and self-reported dietary and drug compliance with optimal control of blood glucose and LDL cholesterol.

## 3. Methods

This was a cross-sectional, descriptive study. Every third patient with T2DM who presented to the outpatient clinic over a period of three months from 1 January 2017 to 31 March 2017 was enrolled after informed consent. Those who reported medical consultation or treatment for medical conditions such as febrile illness, urinary of skin sepsis within the past four weeks, and a history of acute myocardial infarction or stroke within the past three months were excluded. Data on the age, age at onset, and the duration of diabetes were obtained from each patient. Those with a history of hospital admissions for management of myocardial infarction or unstable angina three months before the index visit were considered as having established coronary artery disease (CAD). Body mass index (BMI) was calculated as weight (kg)/height^2^ (m^2^). Self-reported drug and dietary compliance was elicited on a visual analog scale ranging from 0 to 10 (0 = no compliance at all and 10 = complete adherence to prescribed medications and dietary advices given by the dietitian).

All biochemical analyses were performed in the laboratory attached to the Diabetic Center, and the same method of biochemical analysis was used throughout the study period. Overnight fasting venous blood samples were collected to measure HDL-C, LDL-C, serum TG, and glucose. Cholesterol esterase oxidase-peroxidase-amidopyrine method was used to assess serum cholesterol, and for measurement of serum TG, glycerol phosphate oxidase-peroxidase-amidopyrine method was used. For HDL cholesterol, direct method polyethylene-glycol-pretreated enzymes were used. Glycosylated hemoglobin (HbA1c) was estimated using high-performance liquid chromatography (HPLC).

### 3.1. Statistical Analysis

All statistical analyses were performed using the SPSS statistical package. Unpaired *t*-test was used to compare continuous variables, and chi-square test was performed to compare categorical variables in different groups. Logistic regression analysis using age, gender, age of onset, duration of diabetes, self-reported dietary and drug compliance scores, and pill count as independent variables and optimal metabolic control of both HbA1c <% and LDL cholesterol <100 mg/dL as the dependent variable was carried out. *p* < 0.05 was considered statistically significant.

## 4. Results

The mean (SD) age of the total study sample (*n*=207) was 56 (10) years and 55% of them (*n*=113) were males. Descriptive data of the patients participated in the study are shown in Table 1.

Table 2 shows the different medications prescribed to patients participated in the study.

All study participants were prescribed oral hypoglycemic agents and 87% were prescribed statins.

Table 3 shows the percentage attaining the recommended therapeutic targets of glucose, LDL cholesterol, and both parameters.

Table 4 shows a comparison of characteristics in relation to metabolic control in patients with diabetes. Patients with optimal control of both glucose and LDL cholesterol had significantly better self-reported drug and dietary compliance (*p* < 0.05). Logistic regression analysis using age, gender, age of onset, duration of diabetes, self-reported dietary and drug compliance scores, and pill count as independent variables revealed self-reported dietary compliance as significant predictor of optimal control of both blood glucose and LDL cholesterol (OR = 1.3; 95% CI: 1.1 to 1.7).

## 5. Discussion

The most important findings of this study include that less than one-third (30%) of patients with diabetes attained optimal glucose control and less than two-thirds (64%) achieved recommended optimal LDL cholesterol levels. Only one in four patients (23%) with diabetes has achieved optimal levels of both glucose and LDL cholesterol. Out of the demographic and behavioral factors studied, self-reported dietary compliance had significant association with control of both glucose and LDL cholesterol.

Findings from the studies conducted in other Asian countries have revealed similar results especially with the goal achievement in blood glucose. Study of 1520 patients in Saudi Arabia has revealed that HbA1c <% was seen in 40% and LDL <100 mg/dL in 70% [11]. This was a sample with 90% of overweight or obese patients. In a Chinese study with a larger sample size of 2966, HbA1c of <7% was seen in 56% patients [6]. It also revealed that the percentage with optimal HbA1c was different in primary and tertiary care settings (36.2% versus 42.2%). Our study was also conducted in a primary care setting, and the observed glucose control was comparable to the Chinese study. A study from Iran including 2640 patients has reported that optimal glucose and LDL control were seen in 37.4% and 48.9%, respectively [12]. Similar results with regard to blood glucose control have been reported from several Middle Eastern countries with glucose control of HbA1c <7% seen in 40–56% of the patients in a primary care setting [11, 13].

Studies which included blood pressure along with glucose and LDL cholesterol control have revealed that the optimal control of all three factors was seen only in less than one-tenth of the participants. An Iranian study reported that the percentages of patients who had HbA1c <7%, BP <140/90 mmHg, and LDL-C <100 mg/dL were 37.4% (95% confidence interval (CI) 35.6–39.3), 35.3% (95% CI 33.5–37.3), and 48.9% (95% CI 47.0–50.8), respectively. The proportion of patients meeting all three goals was 7.7% (95% CI 6.7–8.8) [12]. A South Korean study has reported a similar percentage of patients (7.8%) achieving optimal control of all three parameters. Another study has revealed that over the duration of one year, the percentage of patients with diabetes achieving the optimal targets has improved from 4.4% in the first year to 14.8% in the second year [14]. A higher percentage (23%) of patients with optimal glucose and LDL cholesterol in our study could be due to factors other than the exclusion of blood pressure in the analysis. Possible explanations include longer mean duration of diabetes (10 years), inclusion of nearly one-third (31%) of patients with established coronary artery disease, and 68% of study participants taking statins. Intensive glucose control in the first group and lipid control measures in the latter two groups could have increased the percentage achieving both glucose and LDL cholesterol goals compared with the reported figures (<10%) elsewhere.

Although several cross-sectional studies in different settings have reported on the percentage of patients with diabetes achieving the recommended glucose, lipid, and blood pressure goals, only a few studies have looked into the demographic and behavioral factors associated with optimal metabolic control. As the control of these parameters can reveal clinically useful and relevant demographic and behavioral correlates, in the present study, we looked into the associations of gender, age at the onset, duration of diabetes, self-reported dietary, and drug compliance and pill count with the optimal control of both glucose and LDL cholesterol. Findings revealed that compared with the group without optimal metabolic control, those achieving the optimal glucose and LDL cholesterol control were older (mean age 58 years versus 54 years), had lesser duration of disease (mean duration 8 versus 10 years), and had better mean scores for self-reported dietary and drug compliance. But out of them, only the latter two behavioral factors, namely, self-reported dietary and drug compliance, showed a statistically significant difference. However, in the logistic regression analysis, only the self-reported dietary compliance score (out of 10) was associated with significant odds ratio (OR: 1.3; 95% CI; 1.1 to 1.7) of achieving optimal glucose and LDL control.

Several demographic and behavioral associations of optimal glucose, lipid, and blood pressure control have been reported in the literature. In a study of electronic database of nearly 300,000 patients with diabetes in Spain, it is reported that both men and women across older age subgroups (>65 years) had longer diabetes duration than younger adults (8.0 versus 5.6 in men and 8.4 versus 6.9 years in women; *p* < 0.001), but had better glycemic control (mean glycated hemoglobin 7.1% versus 7.7% in men and 7.1% versus 7.4% in women; *p* < 0.001) and better combined control of cardiovascular risk factors (*p* < 0.001). Moreover, older patients were more likely to achieve glycemic targets irrespective of having cardiovascular disease [15]. Another longitudinal study from San Diego also reported that the younger age at onset of diabetes was associated with poor glycemic control [16]. However, in our study, although the older patients had better glucose and LDL cholesterol, it was not statistically significant. This could be due to smaller sample size in our study.

The finding of significant association of dietary compliance with optimal control of both glucose and LDL is a very important finding in our study. This has to be considered significant especially because all study participants had been counseled by a dietitian after the diagnosis. Yet, only those with higher self-reported dietary compliance score had a significant optimal control of glucose and lipids. This highlights that advices from the dietitian alone would not result in improving dietary compliance and it is necessary to adhere to the instructions given on diet. In line with our findings, a recent meta-analysis of 773 research reports on behavioral predictors of outcomes in patients with diabetes has revealed that out of the behavioral predictors, dietary adherence was the most significant predictor of HbA1c [17].

Findings of this study have several implications on the management of T2DM and cardiovascular risk factors in developing countries like Sri Lanka. With less than one-third of patients attaining recommended glucose control and less than two-thirds reaching recommended LDL cholesterol targets, more emphasis should be focused on intensification of glucose and lipid control measures among patients with diabetes in Sri Lanka. This could be in the form of updating knowledge on recommended guidelines among primary care physicians and addressing patient's behavioral factors such as improving dietary and drug compliance. Although this study revealed several important findings, it has some limitations. Being a single-center study with small sample size is one limitation. The other important limitation is the methodology used for the assessment of drug and dietary compliance in this study which was a self-reported scale of 0 to 10. This is due to the nonavailability of validated tools for assessment of dietary or drug compliance in the local setting and also practical issues of using such questionnaires for patients attending busy outpatient clinics in the local setting. With the preliminary findings of this study, we recommend that a multicenter study using a validated tool such as a questionnaire designed for this purpose should be carried out.

## 6. Conclusions

We conclude that in this single-center outpatient diabetes clinic in a developing country, attainment of recommended optimal levels of glucose and LDL cholesterol was seen in only 30% and 64% of patients, respectively. Only 23% of patients had archived optimal control of both glucose and LDL cholesterol. Among the demographic and behavioral factors, self-reported dietary compliance score had significant odds of achieving optimal levels of both glucose and LDL cholesterol.

## Figures and Tables

**Table 1:** Descriptive data of the patients with diabetes (*n*=207).

Factor	Percentage
Age (years)^*∗*^	56 (10)
Duration of diabetes (years)^*∗*^	10 (3)
Glycemic control (HbA1c <%)	30
LDL control (LDL <100 mg/dL)	62
Gender (male)	54
Positive family history	59
Presence of established coronary artery disease	31

^*∗*^Given as mean (SD).

**Table 2:** Medications prescribed to the study participants.

Medication	Percentage
Aspirin	22.6
Clopidogrel	14.7
Statin	87.7
Fibrate	2.1
Angiotensin-converting enzyme inhibitor or angiotensin-receptor blocker	57
Oral hypoglycemic agents	100

**Table 3:** Metabolic control with regard to control on HbA1c and low-density lipoprotein.

Factor	Percentage
Optimal glucose control (HbA1c <%)	30
Optimal control in LDL (<100 mg/dL)	62
Optimal control of both glucose and LDL	23

**Table 4:** Comparison of characteristics between patients with and without optimal glucose and LDL cholesterol control.

Variable	Patients with good metabolic control	Patients without good metabolic control	*p* value
Age (years)	58 (9)	55 (11)	0.13
Duration (years)	8 (2)	10 (3)	0.11
Dietary compliance	8 (1)	7 (2)	0.01
Drug compliance	9.8 (1)	9.4 (1)	0.02
Gender (male)	28 (58%)	85 (54%)	0.64
Number of pills	6 (2)	8 (2)	0.12

Data are mean (SD) or *n* (%).

## Data Availability

The data are available as an SPSS file from the corresponding author upon request.

## References

[1] Ramachandran A., Snehalatha C., Ma R. C. (2014). Diabetes in South-East Asia: an update. *Diabetes Research and Clinical Practice*.

[2] UK Prospective Diabetes Study (UKPDS) Group (1998). Effect of intensive blood-glucose control with metformin on complications in overweight patients with type 2 diabetes (UKPDS 34). *The Lancet*.

[3] Genuth S. (2008). The UKPDS and its global impact. *Diabetic Medicine*.

[4] Chamberlain J. J., Herman W. H., Leal S. (2017). Pharmacologic therapy for type 2 diabetes: synopsis of the 2017 American Diabetes Association Standards of Medical Care in Diabetes. *Annals of Internal Medicine*.

[5] Garza L., Dols J., Gillespie M. (2017). An initiative to improve primary prevention of cardiovascular disease in adults with type II diabetes based on the ACC/AHA (2013) and ADA (2016) guidelines. *Journal of the American Association of Nurse Practitioners*.

[6] Bi Y., Zhu D., Cheng J. (2010). The status of glycemic control: a cross-sectional study of outpatients with type 2 diabetes mellitus across primary, secondary, and tertiary hospitals in the Jiangsu province of China. *Clinical Therapeutics*.

[7] Jung J. H., Lee J. H., Noh J. W. (2015). Current status of management in type 2 diabetes mellitus at general hospitals in South Korea. *Diabetes and Metabolism Journal*.

[8] Shrestha S. S., Shakya R., Karmacharya B. M., Thapa P. (2013). Medication adherence to oral hypoglycemic agents among type II diabetic patients and their clinical outcomes with special reference to fasting blood glucose and glycosylated hemoglobin levels. *Kathmandu University Medical Journal*.

[9] Musenge E. M., Michelo C., Mudenda B., Manankov A. (2016). Glycaemic control and associated self-management behaviours in diabetic outpatients: a hospital based observation study in Lusaka, Zambia. *Journal of Diabetes Research*.

[10] Amarasekara A. A., Fongkaew W., Wimalasekera S. W., Turale S., Chanprasit C. (2015). Cross-sectional study of glycemic control among adults with type 2 diabetes. *Nursing and Health Sciences*.

[11] Al-Rowais N. A. (2014). Glycemic control in diabetic patients in King Khalid University Hospital (KKUH)-Riyadh-Saudi Arabia. *Saudi Pharmaceutical Journal*.

[12] Janghorbani M., Papi B., Amini M. (2015). Current status of glucose, blood pressure and lipid management in type 2 diabetes clinic attendees in Isfahan, Iran. *Journal of Diabetes Investigation*.

[13] Al Omari M., Khader Y., Dauod A. S. (2009). Glycaemic control among patients with type 2 diabetes mellitus treated in primary care setting in Jordan. *Primary Care Diabetes*.

[14] Kang A. Y., Park S. K., Park S. Y. (2011). Therapeutic target achievement in type 2 diabetic patients after hyperglycemia, hypertension, dyslipidemia management. *Diabetes and Metabolism Journal*.

[15] Barrot-de la Puente J., Mata-Cases M., Franch-Nadal J. (2015). Older type 2 diabetic patients are more likely to achieve glycaemic and cardiovascular risk factors targets than younger patients: analysis of a primary care database. *International Journal of Clinical Practice*.

[16] Benoit S. R., Fleming R., Philis-Tsimikas A., Ji M. (2005). Predictors of glycemic control among patients with Type 2 diabetes: a longitudinal study. *BMC Public Health*.

[17] Brown S. A., Garcia A. A., Brown A. (2016). Biobehavioral determinants of glycemic control in type 2 diabetes: a systematic review and meta-analysis. *Patient Education and Counseling*.

